# Pericentric inversion in chromosome 1 and male infertility

**DOI:** 10.1515/med-2020-0404

**Published:** 2020-04-20

**Authors:** Ranwei Li, Haitao Fan, Qiushuang Zhang, Xiao Yang, Peng Zhan, Shuqiang Feng

**Affiliations:** Department of Urology, The Second Hospital of Jilin University, Changchun, China

**Keywords:** male infertility, pericentric inversion, chromosome 1, genetic counseling

## Abstract

Pericentric inversion in chromosome 1 was thought to cause male infertility through spermatogenic impairment, regardless of the breakpoint position. However, carriers of pericentric inversion in chromosome 1 have been reported with normal fertility and familial transmission. Here, we report two cases of pericentric inversion in chromosome 1. One case was detected *in utero* via amniocentesis, and the other case was detected after the wife of the carrier experienced two spontaneous abortions within 5 years of marriage. Here, the effect of the breakpoint position of the inversion in chromosome 1 on male infertility is examined and compared with the published cases. The association between the breakpoint of pericentric inversion in chromosome 1 and spermatogenesis is also discussed. Overall, the results suggest that the breakpoint position deserves attention from physicians in genetic counseling as inversion carriers can produce offspring.

## 1 Introduction

A male infertility factor is diagnosed in 50% of infertile couples [1] and affects approximately 4% of men worldwide [2]. Structural chromosomal abnormalities play a major role in perturbing male infertility, resulting in infertility, spontaneous abortion, or the birth of a malformed child [3]. Pericentric inversions are structural chromosomal aberrations caused by 180° rotation of the chromatin segment between these breaks, which result from two breaks on both sides of the centromere [4]. Most individuals with inversion have a normal phenotype and a normal fertility potential. About 12% of the pericentric inversions cause infertility in men [5]. Reproductive risks would be expected in some cases because of the production of chromosomally unbalanced gametes following abnormal meiotic events [6]. Different inversion chromosomes or different breakpoints may lead to different clinical outcomes. Hence, genetic counseling of male carriers of pericentric inversion in chromosome 1 remains a challenge.

Studies have shown that pericentric inversion in chromosome 1 is associated with azoospermia [4,7,8,9]. It was previously considered to cause male infertility through spermatogenic impairment, regardless of breakpoint positioning [10]. With the development of genome sequencing technology, some genes related to spermatogenesis have been found at specific sites on chromosome 1. Bache et al. [11] reported that chromosome 1 could harbor a domain whose integrity is very important for spermatogenesis. However, it has also been reported that carriers of pericentric inversion in chromosome 1 have normal fertility and familial transmission. Sometimes, diagnosis of the condition can be made even before birth [12,13]. The relationship between the specific inversion/breakpoint in chromosome 1 and the clinical outcome requires further clarification.

This study reports on two male cases of pericentric inversion in chromosome 1 and discusses the association between the breakpoint of pericentric inversion in chromosome 1 and male infertility.

## 2 Case report

The subjects of this study were two male carriers of pericentric inversion in chromosome 1. Ethical approval for this study was obtained from the Ethics Committee of the Second Hospital of Jilin University. The patients have provided informed consent for publication of these two cases.

**Figure 1:**
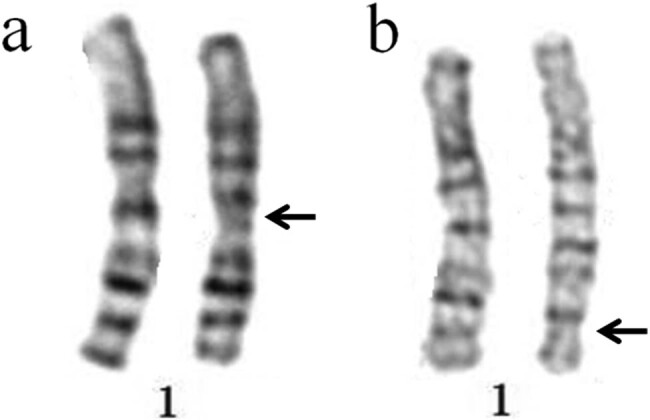
Abnormal karyotypes possessing pericentric inversion in chromosome 1. (a) Karyotype of the first case and (b) karyotype of the second case.

The first case is a 28-year-old man. He underwent cytogenetic detection because fetal chromosomal abnormalities were found during prenatal diagnosis of his offspring during his wife’s second trimester. The karyotype of the fetal chromosome was 46,XY,inv(1)(p13q21). The male carrier had normal appearance and intelligence. The result of G-banding karyotype analysis was 46,XY,inv(1)(p13q21) (Figure 1a). Chromosome preparations and karyotype analysis after G-banding of metaphase chromosomes were carried out according to our previously reported methods [14]. The wife of the carrier underwent a complete gynecological workup, with no abnormalities detected and no history of spontaneous abortion.

The second case is a 31-year-old man of normal phenotype and intelligence. He went to Andrology Outpatient Clinic because his wife experienced two spontaneous abortions within 5 years of marriage. Physical examination revealed the presence of normal-sized testicles with intact vas deferens and normal external male genital organs. Semen analysis showed that semen parameters were within the normal reference range. The result of karyotype analysis was 46,XY,inv(1)(p13q42) (Figure 1b). The wife of the carrier underwent a complete gynecological workup and no abnormalities were detected.

A search for reports on pericentric inversion in chromosome 1 in infertile men was performed using PubMed. The search keywords used were “chromosome 1/pericentric inversion/male infertility.” The case reports of pericentric inversion in chromosome 1 were collected and classified. These included cases of pericentric inversion in chromosome 1 in men of reproductive age and excluded chromosome abnormality in leukemia and other complex structural changes in chromosome. A total of 37 pericentric inversion in chromosome 1 cases were found. The karyotype and clinical findings from the literature analysis are shown in Table 1. These results show that 70.3% (26/37) of the cases presented with spermatogenic disorder.

**Table 1:** Clinical features and karyotype of carriers with pericentric inversion in chromosome 1 reported in the previous literature

Cases	Karyotype	Clinical findings	Reference
1	inv(1)(p36.3q12)	Oligozoospermia	Barros et al. (1986) [16]
2	inv(1)(p36.3q21)	Oligoasthenospermia	Luo et al. (2014) [33]
3	inv(1)(p36.3q43)	Cryptozoospermia	Morel et al. (2007) [34]
4	inv(1)(p36.2q42)	Repeated abortion	Luo et al. (2014) [33]
5	inv(1)(p36.1q32)	Familial miscarriage or stillborn	Johnson et al. (1988) [35]
6	inv(1)(p36q12)	Severe oligozoospermia	Chandley et al. (1987) [10]
7	inv(1)(p36q25)	Azoospermia	Li et al. (2012) [24]
8	inv(1)(p36q25)	Severe oligozoospermia	Zhang et al. (2015) [36]
9	inv(1)(p36q42)	Multigeneration transmission	Honeywell et al. (2012) [12]
10	inv(1)(p36q42)	Recurrent fetal wastage	Fryns and Van Buggenhout (1998) [37]
11	inv(1)(p35q21)	Azoospermia	Rivera et al. (1984) [8]
12	inv(1)(p34q12)	Severe oligozoospermia	Antonelli et al. (2000) [38]
13	inv(1)(p34q23)	Azoospermia	Meschede et al. (1994) [7]
14	inv(1)(p34q23)	Azoospermia	Tóth et al. (1982) [9]
15	inv(1)(p33q25)	Azoospermia	Chandley et al. (1987) [10]
16	inv(1)(p32q12)	Oligoasthenoteratospermia	Gabriel-Robez et al. (1986) [26]
17	inv(1)(p32q21)	Oligoasthenospermia	Luo et al. (2014) [33]
18	inv(1)(p32q21)	Spontaneous miscarriage	Guichaoua et al. (1986) [39]
19	inv(1)(p32q32)	Oligoasthenospermia	Luo et al. (2014) [33]
20	inv(1)(p32q42)	Azoospermia	Chandley et al. (1987) [10]
21	inv(1)(p32q42)	Azoospermia	Batanian and Hulten (1987) [17]
22	inv(1)(p31q12)	Three consecutive spontaneous abortions following the birth of two normal children	Martin et al. (1994) [40]
23	inv(1)(p31q13)	Impaired spermatogenesis	Mierla et al. (2014) [41]
24	inv(1)(p31q43)	Oligozoospermia	Chandley et al. (1987) [10]
25	inv(1)(p22.1q34.1)	Infertility	Young et al. (2019) [42]
26	inv(1)(p22q32)	Azoospermia	Balasar et al. (2017) [4]
27	inv(1)(p22q42)	Oligozoospermia, teratozoospermia	Chantot-Bastaraud et al. (2007) [6]
28	inv(1)(p21q31)	Azoospermia	Kirkpatrick et al. (2012) [43]
29	inv(1)(p13q11)	Recurrent abortions	Sachs et al. (1985) [44]
30	inv(1)(p13q21)	Severe oligozoospermia	Dul et al. (2012) [23]
31	inv(1)(p13q23)	Impaired spermatogenesis	Mierla et al. (2014) [41]
32	inv(1)(p13q23)	Reproductive failure	Gada Saxena et al. (2012) [45]
33	inv(1)(p13q23)	Normal fertility	Uehara et al. (1995) [13]
34	inv(1)(p13q25)	Azoospermia	Giraldo et al. (1981) [46]
35	inv(1)(p11q12)	Oligoasthenospermia	Luo et al. (2014) [33]
36	inv(1)(p13q21)	Prenatal diagnosis of fetus with inv(1)	This study
37	inv(1)(p13q42)	Two spontaneous abortions	This study

## 3 Discussion

Chromosomal abnormalities are one of the most important genetic factors in male infertility. Pericentric and paracentric inversions are found in 0.16% of the men with infertility [15]. Pericentric inversions have a chromosome that is reversed in orientation relative to a normal karyotype, and the rotating segment contains the centromere. In general, men carrying these inversions have a normal phenotype but often show infertility, recurrent pregnancy loss, or an increased risk of their offspring having a congenital anomaly [13]. More attention has been paid to pericentric inversion in chromosome 1, because the disruption of spermatogenesis leads to male infertility regardless of the breakpoint positioning [8,9,10,16,17]. Although several technologies, including Southern blot, fluorescent in situ hybridization, and inverse PCR, are available to detect specific target segments on chromosome, karyotype analysis remains a powerful and cheap technology for clinical practice. In this study, we report two male cases of pericentric inversion in chromosome 1. In one case, conception was normal, and in the other case, the carrier’s wife experienced multiple spontaneous abortions.

In the first case, the karyotype of the male carrier was 46,XY,inv(1)( p13q21). Chromosome inv(1)(p13q21) of the fetus was transmitted from the carrier. Chromosome inv(1)(p13q21) is considered a form of polymorphism according to the International System for Human Cytogenetic Nomenclature [18]. Chromosomal polymorphisms did not appear to have any functional or phenotypic effect and are currently considered a variant of a normal karyotype [19,20]. However, the exact relationship between chromosomal polymorphisms and reproductive disorders is still controversial. Polymorphic variants on chromosomes have often been reported in infertility and recurrent abortions and could play a significant role in infertility [20]. Heterochromatin polymorphism is more frequent in infertile men and should be paid more attention [21]. Polymorphic variants on chromosomes could increase aneuploidies in male gametes and embryos [22]. The karyotype and clinical findings of pericentric inversion in chromosome 1 carriers reported in the previous literature are shown in Table 1. The report of a male carrier with the karyotype inv(1)(p13q21) and severe oligozoospermia is shown in Table 1 [23]. This case has similar breakpoints to those of the first case of this study, but the phenotype is different. This suggests that the role of polymorphic variants with inv(1)(p13q21) in male infertility needs further study.

For the second case, the karyotype of the male carrier was 46,XY,inv(1)(p13q42), and his wife experienced two spontaneous abortions within 5 years of marriage. Spontaneous abortion is possible because spermatozoa with unbalanced chromosomes, produced in meiosis, led to chromosome imbalance in the embryo and repeated abortion. Unfortunately, villus cells of placenta or fetal tissue of the carrier was not genetically tested, so a diagnosis of unbalanced chromosome cannot be ascertained. The two most common types of infertility in male patients are pregestational infertility (exhibit abnormal semen parameters and their partners are not able to conceive) and gestational infertility (partners are able to conceive but have miscarriages) [24]. The second case of this study presented with gestational infertility. However, many men showed pregestational infertility (Table 1), and 70.3% of these cases presented with spermatogenic disorder due to chromosome inv(1) abnormalities. It has been reported that there is no significant relationship between the specific chromosomal breakpoints in chromosome 1 and the degree of spermatogenic failure [7]. However, recent literature indicates that the karyotypic abnormality inv(1) does not always cause infertility. Several male carriers of pericentric inversions in chromosome 1 were detected by amniocentesis (as in the second case of this study) or transmitted through multiple familial generations [13,14,25].

Table 1 shows that each breakpoint of inv(1) may be related to pregestational or gestational infertility and most male carriers exhibited spermatogenic disorders. The exact mechanism of the influence of pericentric inversion on spermatogenesis remains unclear. One hypothesis is that inversions disturb chromosome pairing, synapsis, and recombination during meiosis [4]. However, some scholars reported that spermatogenic failure may not be related to the rearranging autosome or XY pair inverted carriers [26]. The second hypothesis is that inversions cause DNA fragmentation in human spermatozoa and activation of apoptosis [15]. An alternative hypothesis is related to the interference of specific gene function at the breakpoint [4]. By OMIM search, we found 339 genes expressed in testis. The function of these genes in testis is not clear. There are six genes related to human male infertility reported in the literature. Tektin 2 (TEKT2) is located on chromosome 1p34.3, and it is expressed in testis. The loss of TEKT2 results in impaired sperm motility [27]. Spermatogenic failure 21 (SPGF21) gene is mapped on chromosome 1 at 1p22.1, and its mutation leads to acephalic spermatozoa [28]. Cell division cycle 14A (CDC14A) gene is located on chromosome 1p21.2, and its mutation results in high percentage of immotile sperm with abnormal morphology [29]. Sperm mitochondria-associated cysteine-rich protein (SMCP) and ornithine decarboxylase antizyme 3 (OAZ3) genes are mapped on chromosome 1 at 1q21.3. The former is important for the maintenance and stabilization of the crescent structure of the sperm mitochondria [30]. The latter begins to express in the early stage of spermatogenesis and stops in the late spermatid phase [31]. CATSPERE (cation channel, sperm-associated, auxiliary subunit epsilon gene, located on chromosome 1q44) is involved in hyperactivated motility of spermatozoa and male fertility [32]. These genes may be candidate genes for infertility in male carriers of chromosome inv(1).

Interestingly, another concern is that infertile men with inversion chromosomes inherited from their mothers exhibit azoospermia but the carrier’s mother has no indication of subfertility [7,8,16]. Thus, these inversions appear to compromise male but not female fertility, and the mechanism underlying this difference deserves further study. The most frequent rearrangement among the infertile men was inv(1), and among these, pericentric inversions were the most frequent [11]. To explore its patterns of genotype–phenotype correlation, it is necessary to accurately record seminal, endocrine, and histological parameters.

## 4 Conclusion

In conclusion, this study reported two male carriers with pericentric inversion in chromosome 1. These inversion carriers have the possibility of producing healthy offspring. The breakpoint should be assessed by physicians in genetic counseling. The relationship between the breakpoint in chromosome 1 and male infertility deserves further study.
